# From Exposure to Violence between Mother and Her Intimate Partner to Suicidality Experienced by Urban Adolescents in Chicago’s Southside

**DOI:** 10.3390/ijerph18157870

**Published:** 2021-07-25

**Authors:** Jun Sung Hong, Saijun Zhang, Viktor Burlaka, Mieko Yoshihama, Yueqi Yan, Dexter R. Voisin

**Affiliations:** 1 School of Social Work, Wayne State University, Detroit, MI 48202, USA; viktor@wayne.edu; 2 Department of Social Work, University of Mississippi, Oxford, MS 38677, USA; szhang9@olemiss.edu; 3 School of Social Work, University of Michigan, Ann Arbor, MI 48109, USA; miekoy@umich.edu; 4 Biostatistics and Data Support, University of California Merced, Merced, CA 95344, USA; yyan105@ucmerced.edu; 5 Factor-Inwentash Faculty of Social Work, University of Toronto, Toronto, ON M5S 1V4, Canada; dexter.voisin@utoronto.ca

**Keywords:** alcohol, bullying, ethnicity, marijuana, race, tobacco

## Abstract

Although the relationship between exposure to intimate partner violence and youths’ psychological and other wellbeing has been widely studied, there is limited research about how youths’ exposure to violence between mother and her intimate partner may be related to youth psychological wellbeing. The study used a sample of urban adolescents in Chicago Southbound to examine whether youths’ exposure to verbal conflict between mother and her intimate partner is related to their suicidality and whether youth depression and aggression may be in between such a linkage. Our findings indicated that one-third of the youth had suicidal thoughts or suicidal/self-hurting attempts. Youths’ exposure to verbal conflict between mother and her intimate partner was associated with their depressive and aggressive symptoms, and depressive symptoms subsequently were linked to suicidality. The findings also showed that youth depressive symptoms and aggressive symptoms were positively correlated, which may influence their associations with suicidality. We concluded that youth exposure to parental intimate partner violence, even comparatively mild forms such as a verbal conflict between mother and her intimate partner, may increase their risk of suicidality by worsening psychological wellbeing. The findings highlight the importance of tackling youth suicidality risks while accounting for their exposure to intimate partner violence including verbal conflicts between parents.

## 1. Introduction

Adolescence is a developmental period that is characterized as a time of greater vulnerabilities [1], which can be especially stressful due to biological and social changes [2]. While stress is a normal part of adolescent development, consistent exposure to stressful events can increase internalizing behavior problems including suicidal risks, which, according to the Centers for Disease Control and Prevention, is the second-leading cause of death among adolescents (ages 14–18) [3]. For adolescents in low-resourced urban neighborhoods, they are especially vulnerable to suicidal thoughts and behaviors, as socioeconomic disadvantages are shown to increase the risk [4]. Although Whites traditionally have shown higher rates of suicide than Blacks, the racial gap has been narrowing as an increasing number of Black adolescents have reportedly contemplated suicide [5]. Black youth situated in urban neighborhoods that are socioeconomically disadvantaged are especially vulnerable as they have daily exposure to acute and chronic stressors. Among the stressors experienced by urban adolescents, violence in the home is common as it tends to be marked by poverty, crime, and drug-related activities [6]. Additionally, violence involving mothers is an especially serious concern, as children tend to witness violence against mothers more frequently than violence against fathers [7].

While existing research has documented the association between intimate partner violence and suicidal thoughts and behaviors among victims, studies of suicidal ideation (SI) and behavior among those who have been exposed to violence involving parents in the home remain limited. These studies have documented that suicidal thoughts and behaviors also occur among adolescents who frequently witness violence involving a parent in their homes [8,9,10]. Findings from the study of Turner et al. [10], which included a national sample of 1186 youth (ages 10–17), suggest that recent victimization experiences including exposure to violence in the home are precursors to SI. Kim [9] also found that exposure to family violence showed a direct effect on SI in South Korean adolescents. Although years of empirical research had verified that exposure to family violence is a precursor to suicidal behaviors (SB), understanding this relationship is incomplete. Adolescents who are exposed to violence in the family rarely resort to SB immediately. Instead, they are likely to undergo complex pathways, experiencing various psychological, emotional, and behavioral problems before showing suicidal tendencies. The General Strain Theory [11] postulates that adolescents’ “strain” is likely to precede every suicide [12], as suicide is a direct result of adverse emotions, anger, and frustration, resulting from strainful events [13]. The primary aim of the current study is to apply the General Strain Theory to propose and test the pathways from exposure to inter-parental violence to suicidal thoughts and behaviors among a sample of urban adolescents in Chicago’s Southside.

### 1.1. Depressive Symptoms and Aggressive Behaviors

Depressive symptoms and aggressive behaviors are shown to be frequently reported psychological responses to family violence exposures [14,15,16,17]. These psychological responses have also been documented to intensify suicidal thoughts and behaviors [18,19,20,21,22,23]. Depressive symptoms are likely to induce changes in moods, which potentially increases suicidal thoughts and behaviors [24]. Research also suggests common neurobiology of aggressive behavior and suicidality [25], and many individuals who attempted suicide show significantly higher scores for lifetime and trait aggression [26]. Existing studies have documented that adolescents with high levels of aggression tend to engage in self-injury [27] and SI [28]. In the study of Miotto et al. [29], 30.8% of females and 25.3% of males with aggressive tendencies reported SI. This connection between suicidality and aggression is particularly prominent in clinical research with persons who have a history of attempted suicide [30]. Similarly, youth with suicidal tendencies may be more likely to display aggressive behaviors [30]. Additionally, depression, which is a significant risk for SB, shows an important linkage with aggressive behaviors [30].

Research further shows a bidirectional relationship between depressive symptoms and aggressive behaviors, which might also interact in the association between family violence exposure to suicidality. Although depressive symptoms and aggressive behaviors have been presumed to be mutually exclusive due to differences in energy requirements and blame orientation [31], studies report that individuals with depressive symptoms have an elevated risk for aggressive tendencies [31]. In one study, high levels of aggressive behaviors were found in adolescents with major depressive disorders [31]. In another study, the mean score of aggressive behaviors in depressed females was significantly higher than in non-depressed females [32]. Moreover, aggressive behaviors are positively correlated with depressive symptoms [33,34,35]. As aggressive behaviors worsen over time, so may depressive symptoms [36], which suggests a reciprocal relationship between the two.

### 1.2. The Current Study

The present study tests the potential pathways from exposure to verbal conflict between mother and her intimate partner to suicidality among a sample of urban adolescents in Chicago’s Southside. Guided by the General Strain Theory, we hypothesize that (a) exposure to verbal conflict between mother and her intimate partner will be directly associated with suicidality, (b) depressive symptoms will mediate the association between exposure to verbal conflict between mother and her intimate partner and suicidality, and (c) there will be a reciprocal association between depressive symptoms and aggressive behaviors and between aggressive behaviors and suicidality.

## 2. Materials and Methods

Twenty trained graduate research assistants recruited prospective study participants from 25 high schools in Chicago’s Southside in April 2016. The sample for the study lived together or had close contact with their mother who was in a romantic relationship. The research assistants distributed consent forms to 673 students (ages 14 to 19), of whom 80% were Black. For those younger than 18 years of age, both consent from the parents and written assent from the students were obtained before the students filled out the self-administered survey. Students 18 or older provided written consent. The paper-and-pencil surveys were administered in a small school auditorium during class time, and the participants were asked to complete the survey within 40 min. The participants were informed that their participation was strictly voluntary, and they had the option to withdraw from the study at any time. Upon completion of the survey, participants received $10. The current study focused on 570 youth who were 14 years and older. There was a small amount of missing data across the variables, with the maximal missing of 23 out of the 570 participants for one item measuring youths’ depressive symptoms. The University Institutional Review Board of the last author, the local school district, and the regional school officials approved the study procedures.

### Measures

Youths’ exposure to verbal conflict between mother and her intimate partner was assessed with two items derived from the Revised Conflict Tactics Scale-2 [37]. Youth were asked the frequency of exposure to verbal conflict between their mother and her partner throughout their lifetime with the following two questions: “How many times have you witnessed your mom argue with a partner?” and “How many times have you heard your mom and a partner yelling or screaming at each other, or one screaming or yelling at the other?” Response options ranged from 0 times (0) through 6 times or more (6) on a 7-point Likert-type scale. Responses from the two items were summed to form a scale ranging from 0 to 12, with larger values indicating more frequent exposure to violence between the mother and her intimate partner (α = 0.92).

Youth depressive symptoms was a latent variable measured with four items derived from the Youth Self-Report survey by Achenbach [38]. Youth were asked to rate the truthfulness of various statements concerning their behavior in the past six months. The included items are “There is very little that I enjoy”, “I feel that no one loves me”, “I feel worthless or inferior”, and “I feel too guilty.” Response options are on a 3-point Likert-type scale including not true (0), somewhat or sometimes true (1), and very true or often true (2). Because of the scarcity of responses in the category of (2), response values of (1) and (2) were combined and indicated by a value of 1 (α = 0.61).

Youth aggressive behavior was a latent variable measured with four items derived from the Youth Self-Report survey by Achenbach [39]. Youth were asked to rate the truthfulness of various statements concerning their behavior in the past six months. The included items are: “I destroy things that belong to others”, “I get in many fights”, “I physically attack people”, and “I threaten to hurt people”. Response options are on a 3-point Likert-type scale including not true (0), somewhat or sometimes true (1), and very true or often true (2). Because of the scarcity of responses in the category of (2), response values of (1) and (2) were combined and indicated by a value of 1 (α = 0.6).

Suicidality was a variable consisting of two items derived from the Youth Self-Report survey by Achenbach [39]. Youth were asked to rate the truthfulness of various statements concerning their behavior in the past six months. The included items are “I deliberately try to hurt or kill myself” and “I think about killing myself”. Response options are on a 3-point Likert-type scale including not true (0), somewhat or sometimes true (1), and very true or often true (2). Positive responses (e.g., responses with value 1 or 2) were rare for the item “I deliberately try to hurt or kill myself”. The variable suicidality was created based on the item “I think about killing myself”. If responses for the item “I deliberately try to hurt or kill myself” had a value of 1 or 2, they will be coded as 2 in the variable suicidality.

Several variables were included as covariates in the structural equation modeling (SEM), including age (14 to 18), sex (1 = female and 0 = male), race (Black = 1 and other = 0), two-parent family (1 = yes and 0 = no), and whether receiving free lunch (1 = yes and 0 = no or non-response).

## 3. Results

**Figure 1:**
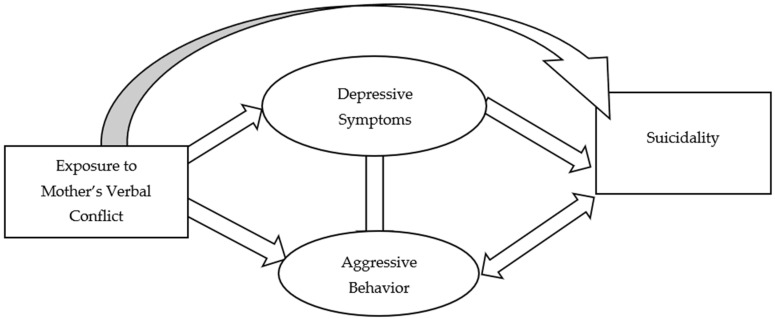
Conceptual framework.

Descriptive analyses were conducted to illustrate the characteristics of the study participants and items of key measures. Structural equation modeling (SEM) was used to test hypotheses based on the conceptual framework as shown in Figure 1. Models such as item correlations were adjusted based on model fit indices to improve model performance.

Lavaan in *R* was used for the SEM [39]. The SEM was run with 500 bootstrap samples. Bootstrapping uses random sampling with replacement techniques to obtain robust standard errors and bias-corrected estimations, which is particularly recommended for the estimation of mediating effect estimation [40]. We controlled for youths’ age, sex, race, family structure, and whether receiving free lunch as covariates in the predictive models involved in the SEM.

We used common model fit indices to assess the model fit for the SEM. The chi-square model fit test with a *p*-value greater or equal to 0.05, Tucker–Lewis Index (TLI) and Comparative Fit Index (CFI) of about 0.95 or greater, root mean squared error of approximation (RMSEA) < 0.06, and standardized root mean squared residual (SRMR) < 0.08 suggest a good model fit [41]. Because the chi-square test result is subject to sample size and is regarded as less reliable, other indices are regarded as more robust criteria [42].

### 3.1. Descriptive Statistics

Table 1 presents the overall characteristics of the study participants and means and standard deviations for key study measures. On average, youth were 16.07 years old, 62% were female, most identified as Black (96%), 28% were in two-parent families, and 59% received free lunch. Youth rating of two indicators of exposure to verbal conflict between mother and her intimate partner was 3.23 and 3.38 in the range of 0 to 6 and the sum for the compound scale was 6.62 in the range of 0 to 12, which indicates that on average youth had witnessed their mother arguing with a partner or hearing them yelling at each other 6 or more times.

For the youth depressive symptoms measure, ratings of the indicators ranged from 0.12 to 0.26 in the range of 0 to 1. For the youth aggression measure, ratings of the indicators ranged from 0.13 to 0.38 in the range of 0 to 1. In both cases, the value 0 indicated that youth having no depressive symptoms, and 1 indicated that youth having depressive symptoms. For the measurement of youth suicidality, the mean rating was 0.39 in the range of 0 to 2, which was slightly approaching agreeing having thought of killing self or conducting suicide or self-hurting (see Table 1). We also examined the suicidal thoughts and SB or self-hurting more closely before combining the two items. In the sample, 31.5% of the youth reported thinking of killing themselves as being at least somewhat or sometimes true, and 2.5% of the youth reported deliberately trying to kill or hurt self as being at least somewhat or sometimes true. Together 33% of the youth ever thought of or tried to kill or hurt themselves (extra analyses are not shown).

### 3.2. SEM Analyses

**Figure 2:**
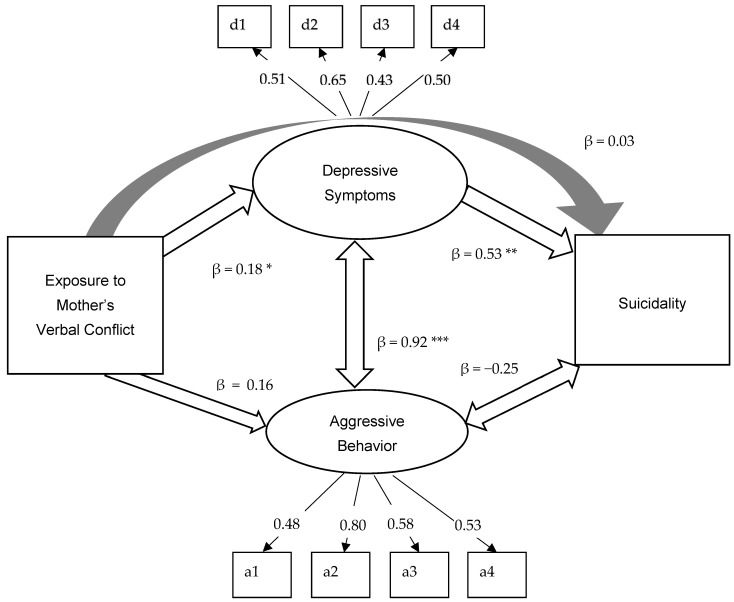
Results from the SEM Analyses. Note: Model fit: χ2 (df = 47) = 87.1, *p* = 0.002; CFI = 0.93; TLI = 0.95; RMSEA = 0.04; SRMR = 0.08. The model controlled for adolescents’ age, sex, race, family structure, and whether receiving free lunch. * *p* < 0.05 ** *p* < 0.01 *** *p* < 0.001.

As shown in Figure 2, the model fit the data well, *χ^2^* (*df* = 47) = 87.1, *p* = 0.002; CF1 = 0.93; TLI = 0.95; RMSEA = 0.04; SRMR = 0.08). Standardized factor loadings for depressive symptoms ranged from 0.43 to 0.65, and standardized factor loadings for aggressive behaviors ranged from 0.48 to 0.80.

Youths’ exposure to verbal conflict between mother and her intimate partner was related to their depressive symptoms (*β* = 0.18, *p* = 0.034), and youth depressive symptoms were related to youth suicidality (*β =* 0.53, *p* < 0.001). The indirect effect from youth exposure to verbal conflict between mother and her intimate partner to youth suicidality via youth depressive symptoms was not statistically significant (*β =* 0.1, *p* = 0.08), and the direct effect from the exposure to suicidality was not statistically significant either (*β =* 0.03, *p* = 0.681). However, the total effect in the aforementioned paths was statistically significant (*β =* 0.13, *p* = 0.03).

On the other hand, the association from youth exposure to verbal conflict between mother and her intimate partner to youth aggressive behaviors was statistically significant (*β =* 0.16, *p* = 0.014). In the two pairs of examined correlations, youth depressive symptoms and youth aggressive behaviors were significantly correlated with each other (*β =* 0.92, *p* < 0.001), but youth aggressive behaviors and suicidality were not (*β =* −0.25, *p* = 0.16) (see Figure 2).

## 4. Discussion

The present study used the General Strain Theory to examine the association between youths’ exposure to verbal conflict between mother and her intimate partner and youth mental health and suicidality in a sample of urban adolescents in Chicago’s Southside. The study contributes to the scarce literature on urban adolescents’ exposure to violence between parents in the United States. Our hypothesis that exposure to verbal conflict between mother and her intimate partner is directly associated with suicidality was not supported by data. However, youth exposure to verbal conflict between mother and her intimate partner was linked with increased aggressive and depressive symptoms. Furthermore, the suicidal risk was higher among youths who reported higher depressive behaviors.

These findings are inconsistent with the previous literature that family violence plays a significant role in adolescent SB [8,43]. Unlike the work in [8] and that by Roland et al. [43], which measured serious forms of interpersonal violence between parents and demonstrated a significant association with adolescents’ SB, our data considered adolescent’s perceptions of a milder form of interparental violence, verbal conflict. Our study did not find exposure to verbal conflict between mother and her intimate partner to be significantly associated with suicidality, which might suggest that verbal conflict involving mothers may be perceived to be “less serious” and may not impact adolescents’ emotional and behavioral well-being as harmfully as those serious forms.

We also tested whether depressive symptoms would mediate the association between exposure to verbal conflict between mother and her intimate partner and suicidality. Although exposure to verbal conflict between mother and her intimate partner was significantly associated with youths’ depression, and youths’ depression was significantly related to suicidality, depression has not mediated the association between exposure to verbal conflict between mother and her intimate partner and suicidality. Several explanations can explain this result: Exposure to verbal conflict between mother and her intimate partner may not be as traumatic for children as some other types of interparental violence that involve, for example, lacerations or gun violence that can result in death or imprisonment of the caregivers. Our moderate community sample of 570 students may also limit the detection of the associations. Depression might demonstrate its mediating effect between exposure to parental verbal abuse and SB in larger populations. Another explanation could be the presence of the other unobserved mediator or mediators that were not included in our study. Future studies may test, for example, whether exposure to verbal conflict between mother and her intimate partner leads to increased self-blame and a sense of guilt [8] as well as thwarted belongingness and perceived burdensomeness [44] in some students that can increase their risk of resorting to suicide. According to Joiner [45], three factors that mark those most at risk of suicide include a feeling of being a burden on a loved one, a sense of isolation, and a learned ability to harm oneself. Alternatively, future research may test whether the knowledge and use of coping skills can mediate the relationship between exposure to stressful events such as a verbal conflict between mother and her intimate partner and engaging in SB [46].

We also tested the reciprocal relationships between depressive symptoms and aggressive behaviors, and the reciprocal relationship between aggressive behaviors and suicidality. Consistent with prior research, children who experienced depressive symptoms were more likely to report aggressive behaviors. The association between internalizing and externalizing behaviors can be observed in children as young and 3–5 years of age [47]. Children are likely to be maltreated in families characterized by violence and conflicts [48] and have a higher risk to develop emotional dysregulation [49]. Importantly, children who are raised in dangerous and unpredictable settings often adapt their behaviors to fit in the local context. Our finding that depressive symptoms co-occur with aggressive symptoms is consistent with the Adaptive Calibration Model of individual differences in stress responsivity [50]. According to this model, the different adaptive child responses to the developmental contexts can range from being sensitive and buffered to being vigilant (agonistic or withdrawn), or even unemotional depending on the degree of perceived environmental stress and support.

Despite prior studies, which show a significant association between aggressive behavior and SB [29,30,31], our hypothesis that aggressive behaviors would correlate with suicidality has not been supported. A possible reason may be that our measurement of aggression with four items on a three-point scale is less precise than those used in other studies and thus could not detect its correlation with suicidality.

In summary, our study has explored the association between exposure to verbal conflict between mother and her intimate partner and adolescent mental health and suicidality in impoverished Chicago neighborhoods using the General Strain Theory. The study has documented the positive association between exposure to verbal conflict between mother and her intimate partner and youth depression and aggression. In addition, children who reported more symptoms of depression were more likely to engage in SB. These findings highlight the complexity of risk associated with youth suicide. Suicide prevention programs should address both individual and family risk factors related to SB.

### 4.1. Limitations and Implications for Future Research

Although this study makes a unique contribution to the existing literature on the association of exposure to verbal conflict between mother and her intimate partner with youth mental health and suicidality among urban communities, a few limitations of this study must be mentioned. First, this study mainly relies on a cross-sectional design. Given exposure to verbal conflict between mother and her intimate partner at home is often chronic, longitudinal studies are needed to examine the long- versus short-term adverse effects of family violence on youth mental health and suicidality among Black communities. Second, due to data limitation, exposure to verbal conflict between mother and her intimate partner measured verbal conflict and did not assess youths’ exposure to other forms of violence in the family or direct experiences of abuse or neglect, both of which are highly correlated with exposure to parental intimate partner violence [51]. Forthcoming research should explore how different types of interparental violence differ in affecting youth mental health conditions. Third, the survey was administered only in the high schools in Chicago’s Southside. The generalization of the study can be limited as indigenous culture, community environment and social support vary and can influence youth mental health as well as how they perceive interparental violence. Fourth, although “receiving free lunch” was controlled for in the analyses, we did not include a full measure for the socioeconomic statuses. Researchers are advised to consider measures for socioeconomic status, such as parents’ educational attainment and occupational status. Fifth, the construct for youths’ exposure to verbal conflict between mother and her intimate partner, depressive symptoms, aggressive behavior, and suicidality were assessed with a limited number of items. The period assessed for youths’ exposure to verbal conflict between mother and her intimate partner appears to be too long for expecting valid information. Future studies might consider more valid measures, such as the Center for Epidemiology Studies-Depression Child to assess youths’ depressive symptoms. Sixth, also related to the measures, the study only examined verbal forms of conflict between mothers and their intimate partners, which cannot be controlled for other forms of violence between parents, which tend to co-occur. It is imperative that studies include various forms of violence between parents, which would include verbal and physical forms of violence. Seventh, given the rarity of self-reported suicide attempts (SA) in our community samples, we chose to combine SI with suicide attempt items. Larger-scale studies are needed to differentiate the effects of family violence on SI from SA among Black communities. Eighth, studies building on this finding might investigate other relevant mediators or moderators, such as the function of personality and how they might be associated with exposure to intimate partner violence between parents and SI of adolescents [52]. Ninth, the study relied on data collected in 2016, which might be somewhat dated, but still relevant. Exposure to conflict and violence between parents and adolescent suicidality remains a serious problem in impoverished neighborhoods, and our findings have implications for research and practice. Last, future research should consider exploring the possible role of epigenetics in SI among adolescents whose parents are involved in violence [53].

### 4.2. Implications for Practice

The National Survey of Children’s Exposure to Violence reports an alarmingly high rate of exposure to family violence among children, especially among youth aged 14–17; one in four youth aged 14–17 (25.4%) is exposed to psychological or emotional abuse between parents, and one in ten, to verbal abuse between parents [54]. Our findings (in which Black adolescents were over-represented in the study sample) calls for identification and assessment tools that are culturally informed. Such assessment and intervention must pay particular attention to SI. While aggressive behavior was not directly associated with suicidality, its strong association with depressive symptoms, which was associated with suicidality deserves special attention. In clinical evaluations of youth, practitioners should pay attention to the meaning and contributing factors of aggressive behaviors and assess for histories of exposure to family violence, depressive symptoms, and SI. Additionally, the pivotal role of familial relationships is an important consideration in the assessment. Practitioners need to identify protective factors within the family context that potentially reduce adolescents’ risk of SI. In their research on suicidal patients, Costanza et al. [52] found that family served a salient protective role. Moreover, at the mezzo levels, schools must develop systems of care for students exhibiting aggressive behaviors. Too often aggressive behaviors, especially in low-income youth of color, are subjected to disciplinary sanctions such as suspension and expulsion from school [55,56]. Out-of-school suspension is associated with negative outcomes, such as a sense of alienation, poor academic achievement, delinquency, and substance use [57,58,59]. Schools serve as an important resource for youth in need; “commitment to school” is one of the few factors found to “buffer the effects of exposure to specific risks for violence” [60] (p. xii) and is a protective factor against antisocial/delinquent behaviors in youth. Instead of driving youth exhibiting aggressive behavior out of school, knowing that aggressive behaviors might be related to the conflict in the family and/or depressive symptoms, timely assessment and treatment should be provided.

## Figures and Tables

**Table 1:** Descriptive statistics of the study sample (*n* = 570).

Variable	*M*/%	*SD*	Min	Max	α
Age	16.07	1.06	14	18	
Female	0.62	0.49	0	1	
Black/African American	0.96	0.2	0	1	
Two-parent family	0.28	0.45	0	1	
Receiving free lunch	0.59	0.49	0	1	
Youths’ exposure to verbal conflict scale	6.62	4.81	0	12	0.92
How many times have you witnessed your mom argue with a partner?	3.38	2.47	0	6	
How many times have you heard your mom and a partner yelling or screaming at each other, or one screaming or yelling at the other?	3.23	2.54	0	6	
Youth depressive symptoms scale	0.18	0.23	0	1	0.61
d1. There is very little that I enjoy	0.26	0.44	0	1	
d2. I feel that no one loves me	0.12	0.33	0	1	
d3. I feel worthless or inferior	0.2	0.4	0	1	
d4. I feel too guilty	0.13	0.34	0	1	
Youth aggressive behavior scale	0.25	0.26	0	1	0.6
a1. I destroy things that belong to others	0.13	0.34	0	1	
a2. I get in many fights	0.38	0.49	0	1	
a3. I physically attack people	0.21	0.4	0	1	
a4. I threaten to hurt people	0.27	0.44	0	1	
Suicidality	0.39	0.6	0	2	

## Data Availability

The study did not report any data.
